# Clinical and Demographic Characteristics of Patients With a New Diagnosis of Carriage or Clinical Infection With Carbapenemase-Producing *Enterobacterales*: A Retrospective Study

**DOI:** 10.3389/fpubh.2021.616793

**Published:** 2021-02-05

**Authors:** Assaf Adar, Hiba Zayyad, Maya Azrad, Kozita Libai, Ilana Aharon, Orna Nitzan, Avi Peretz

**Affiliations:** ^1^The Azrieli Faculty of Medicine, Bar-Ilan University, Safed, Israel; ^2^Infectious Disease Unit, The Baruch Padeh Medical Center, Tiberias, Israel; ^3^Clinical Microbiology Laboratory, The Baruch Padeh Medical Center, Tiberias, Israel

**Keywords:** carbapenem-resistant *Enterobacterales*, carbapenemase-producing *Enterobacterales*, antibiotic resistance, Israel, carrier

## Abstract

**Background:** To examine the clinical, demographic, and microbiologic characteristics of new rectal carbapenemase-producing carbapenem-resistant *Enterobacterales* (CP-CRE) carriers vs. those with a clinical infection, hospitalized at Padeh-Poriya Medical Center between 2014 and 2017 and to examine the susceptibility profiles of isolates from clinical infections.

**Methods:** In this retrospective, chart analysis, demographic and clinical data were collected from medical charts of 175 adult patients with either new- onset carbapenemase-producing *Enterobacterales* (CPE) carriage or clinical CPE infection. Collected data included age, ethnic group, place of residence, hospitalizations in the past 90 days, and 30-day mortality. Microbiological analyses considered bacterial genus, molecular resistance mechanism and antibiotic susceptibility.

**Results:** A significantly higher percentage (42.4%) of CPE carriers were long-term care facility residents, and had been recently hospitalized (56.3%), as compared to patients with clinical CPE infection (29.2 and 45.9%, respectively). Additionally, we noted a high (58.3%) acquision of CPE in our hospital. The most common bacterial isolate was *K. pneumoniae* and the most common resistance mechanism was *Klebsiella pneumoniae* (*K. pneumoniae)* carbapenemases (KPC). High susceptibility rates to amikacin and chloramphenicol were also noted.

**Conclusions:** This study reaffirmed the importance of CPE screening and infection control measures. The observed antibiotic susceptibility profile suggests amikacin and chloramphenicol as potential treatments for CPE infection.

## Introduction

Antibiotic resistance is one of the most significant challenges of twenty-first century medicine. As the diversity of resistant strains and resistance mechanisms continues to grow, so does the carrierage rate in both hospitalized patients and in the community, raising considerable concern among researchers, clinicians and international health-care systems (1). Carbapenem-resistant *Enterobacterales* (CRE) are of particular importance due to their diverse and extensive resistance, frequently even to extended-spectrum antibiotics (2). CREs are classified as carbapenemase producers (CPE) or non-producers (non-CP CRE), with the most common carbapenemase genes being KPC (Ambler Class A) and OXA-48-like (OXA-48) (Ambler class D) carbapenemases in the developed world, and Verona integron-encoded metallo-β-lactamase (VIM), New-Delhi metallo-β-lactamase (NDM), and IMP-type metallo-β-lactamase (IMP) in the developing world (3). Global dissemination of CPE is now occurring at an alarming pace and is complicated by limited treatment options, which currently include polymyxins, tigecycline, aminoglycosides and, in some cases, high-dose carbapenems (4), all of which are frequently ineffective and associated with a myriad of adverse effects. CPE infections can include urinary tract infection (UTI), intra-abdominal infections, pneumonia (especially in ventilated patients), sepsis, skin and soft tissue infections, and surgical site infections. They are associated with a 3 to 6-times higher mortality rate as compared to non-CP CRE infections (1, 5), and possess a higher potential to spread to other patients (6). The main reservoirs for CPE spread are asymptomatic carriers who transmit these bacteria in long-term care facilities (LTCF) and hospitals in the developed world and feco-oral contamination in the developing world (7), which carries increased risk for active infection and is associated with increased mortality (8).

In recent years, Israel has seen several outbreaks of CPE, which affected major medical centers nationwide, with a peak in 2007, mainly due to clonal strains of *K. pneumoniae* bearing KPC-2 and KPC-3 carbapenemases (9). The outbreaks prompted a national program instating mandatory reporting, screening, and isolation of carriers and infected patients in a designated area of the hospital, assigning designated medical staff, and heightening implementation of sanitation and hand hygiene (8). Today, screening is conducted by rectal swab in high-risk patients such as LTCF residents, patients with recent prior hospitalization, intensive care unit (ICU) patients, and patients transferred between wards inside the hospital.

To identify patient characteristics that may contribute to the development of clinical CPE infection and shed light on the effectiveness of the current prevention control strategies, this study reviewed the clinical and demographic profiles of patients who had a CPE carrier diagnosis or a clinical infection with CPE, and hospitalized in a small hospital in northeastern Israel, between 2014 and 2017.

## Methods

### Study Design

Clinical and demographic data were collected from the medical records and microbiological lab reports of all adult (>18 years) rectal asymptomatic CPE carriers or adults with a clinical infection with CPE, registered at the Padeh-Poriya Medical Center in northern Israel, between January 2014 and December 2017. Our medical center is a small hospital (350 beds), with small ICU and surgical units, and without post-transplantation unit.

Exclusion criteria included patients with a known diagnosis of CPE carriage\infection in the 90 days before the new CPE diagnosis at the Padeh-Poriya Medical Center.

The study was approved by the Padeh-Poriya Medical Center institutional ethics committee, approval no. POR-0031-17.

### Bacterial Isolates

#### Bacterial Isolates of CPE Carriers

As part of the hospital's infection control program, CRE screening is performed in patients who were hospitalized in the preceding 6 months, are from a LTCF, were transferred from another hospital, are being hospitalized for more than 1 month, or were transferred between hospital wards after 72 h, to and from the ICU, or to the hospital rehabilitation unit after more than 7 days. A rectal swab was collected from each patient and sent to the laboratory. As part of the routine microbiology laboratory practice, the swabs were inoculated in Brain-Heart infusion broth (Hy-Laboratories Ltd., Rehovot, Israel), supplemented with meropenem (10 mg) disk (BD Diagnostics, Sparks, MD) and then incubated at 37°C, for 24 h. Following incubation, the swabs were inoculated on BD BBL™ CHROMagar™ CPE (BD Diagnostics, Sparks, MD) medium, that was subsequently incubated for another 24 h, at 37°C. Following incubation, the agar plate was screened for the presence of pink and blue colonies, using matrix-assisted laser desorption ionization-time of flight (MALDI-TOF) technology (Bruker Daltonics, Bremen, Germany). If the identified species belonged to the Enterobacterales, then the colonies were tested using the β CARBA kit (Bio-Rad Laboratories Ltd., Rishon Lezion, Israel), which detects strains with decreased susceptibility to carbapenems. If the test yielded a positive result, the suspected colonies were further analyzed by the Xpert® Carba-R (Cepheid, Sunnyvale, CA, USA), a PCR assay that detects the five most prevalent carbapenemases (KPC, NDM, VIM, OXA-48, and IMP).

#### Bacterial Isolates of Clinical Infection

Various clinical samples (including urine, blood, peritoneal fluid, wounds, tissue, and endotracheal tube sample) were collected from patients hospitalized at the medical center and sent to the laboratory. As part of the routine practice of the microbiology laboratory, each sample was inoculated on several media. Following incubation, suspected colonies were identified using the MALDI-TOF technology (Bruker Daltonics, Bremen, Germany) and then antibiotic susceptibility testing was performed according to the bacterial and infection type, using the disk diffusion method (Kirby Bauer) in accordance with the European Committee on Antimicrobial Susceptibility Testing (EUCAST) 2014–2017 guidelines. An isolate belonging to the *Enterobacterales* family and with a decreased susceptibility to carbapenems, was tested with the β CARBA kit (Bio-Rad Laboratories Ltd., Rishon Lezion, Israel) and the Xpert® Carba-R (Cepheid, Sunnyvale, CA, USA), as described in the previous section.

### Data Collection

The data collected included demographic data such as age, ethnic group, residential environment (home vs. LTCF), previous hospitalizations (in the past 90-days) and clinical data, such as cause of hospitalization, duration of hospitalization, 30-day mortality and type of acquisition (nosocomial vs. community; according to the Israeli Ministry of Health guidelines, community-acquired infection is defined as acquisition within the 48 h from hospitalization). Collected microbiological data included bacterial genus, resistance mechanism and antibiotic susceptibility.

### Statistical Analysis

Microsoft Excel 2016 (Microsoft, Redwood, WA) was used for data capture. The Chi-squared test was applied to analyze differences in categorical variables (percent) between sub-groups. The analysis of variance (ANOVA) model was applied to analyze differences in quantitative (continuous) variables between sub-groups. All tests were two-tailed, and a *p*-value of 5% or less was considered statistically significant. The data were analyzed using SAS®, version 9.3 (SAS Institute, Cary North Carolina).

## Results

### Clinical and Demographic Data

**Figure 1:**
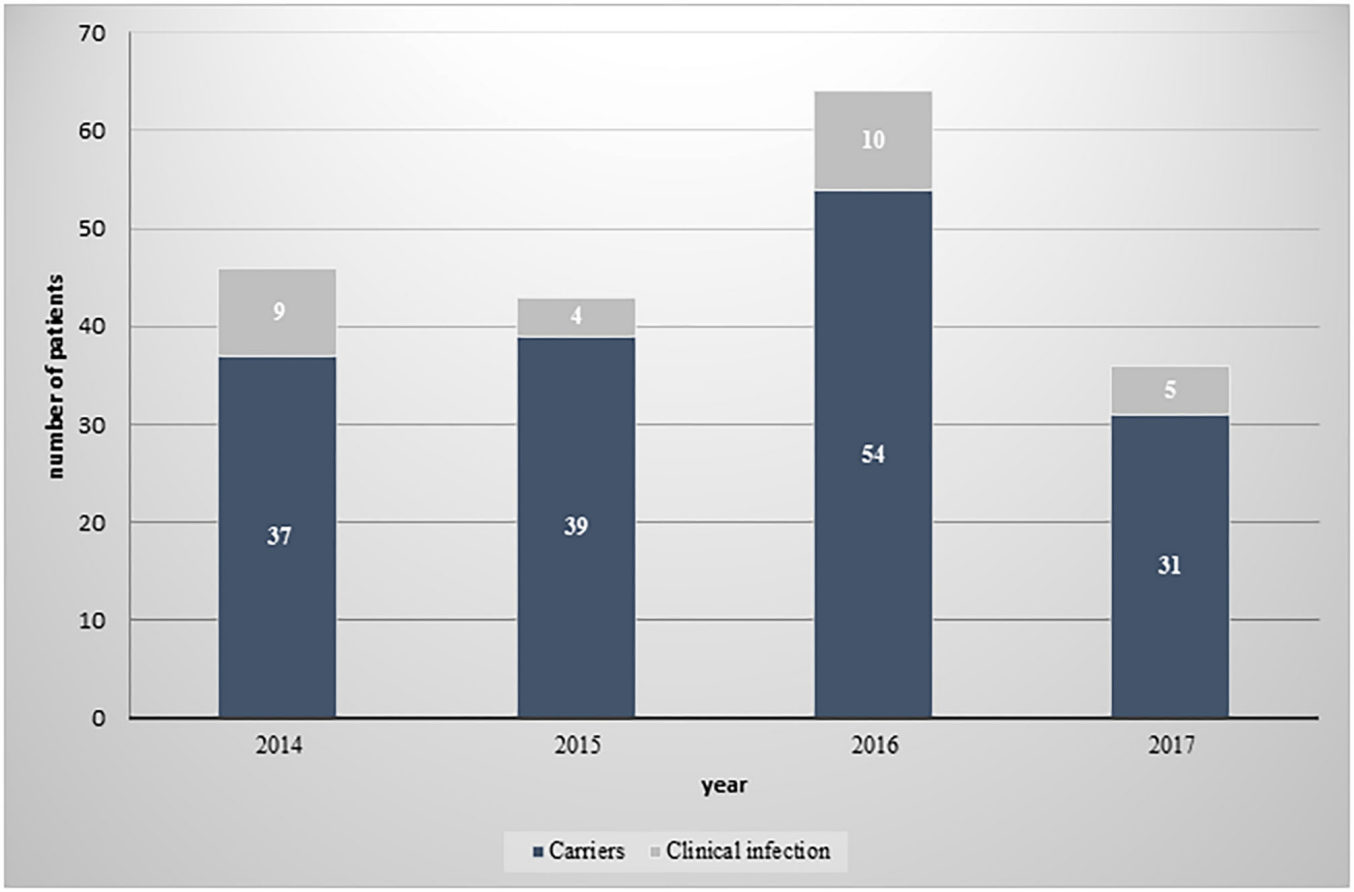
Distribution of CPE isolates between carriers and patients with clinical infection, by year.

Of the 12,984 CPE tests performed on samples collected for screening or clinical diagnostic purposes from patients hospitalized in the medical center between 2014 and 2017, 175 (1.34%) were found positive. Among these, 24 (13.7%) patients were diagnosed with a clinical infection caused by CPE, as detected in urine cultures (11 [39%] of clinical infection isolates), blood cultures (6 [22%]) or other biological samples (peritoneal fluid, wound culture, tissue culture, and endotracheal tube culture; 11 [39%]). The remaining 151 (86.2%) patients were CPE carriers who were surveyed by rectal swabs. The numbers of CPE carriers and of patients with clinical infections per study year, are displayed in Figure 1.

A summary of the demographic characteristics of the patient carriers vs. patients with clinical infection can be viewed in Table 1. A significantly higher percentage of LTCF residents (42.4%) in the carrier group (*p* < 0.001), in contrast to a significantly higher percentage of community residents in the clinical infection group (70.8%) (*p* < 0.001). In addition, nosocomial acquisition of CPE occurred in 41.7% of all patients and was significantly more common in the clinical infection group (58.3%) (*p* < 0.001). A significant majority of patients (54.7%) had been hospitalized in the 3 months prior to their initial CPE diagnosis, most of whom had been hospitalized at the Padeh-Poriya Medical Center (45.1%) and 9.7% in other medical centers in Israel (*p* < 0.001).

**Table 1:** Demographic and baseline characteristics of patients with CPE carriage or clinical CPE infection.

**Characteristics**	**Carriers** **(*N* =151)**	**Clinical infection** **(*N* = 24)**	**All patients** **(*N* = 175)**	***p*-value**
Mean age (years)	69.3 (21.0–103.0)	73.1(30.0–91.0)	69.8 (21.0–103.0)	0.34
**Ethnic group**, ***n*** **(%)**				
Jewish	114 (75.5)	16 (66.7)	130 (74.3)	0.35
Non-Jewish	37 (24.5)	8 (33.4)	45 (25.7)	
**Residential environment**, ***n*** **(%)**				
Community	87 (57.6)	17 (70.8)	104 (59.4)	<0.001^*^
Long-term care facility (LTCF)	64 (42.4)	7 (29.2)	71 (40.5)	
Duration of hospitalization (days), mean (range)	18.0 (2–154)	23.5 (1–76)	18.7 (1–154)	0.29
30-day mortality	28 (18.5)	6 (25)	33 (18.9)	** <0.001**
Hospitalization in the 3 months before diagnosis, *n* (%)	85 (56.3) 69 in PPMC	11 (45.9) 10 in PPMC	96 (54.7)	** <0.001**
Acquisition during current admission, *n* (%)	59 (39.1)	14 (58.3)	73 (41.7)	** <0.001**
**Ischemic heart disease**, ***n*** **(%)**				
Yes	56 (37.1)	10 (41.7)	66 (37.7)	0.66
No	95 (62.9)	14 (58.3)	109 (62.8)	
**Diabetes**, ***n*** **(%)**				
Yes	70 (46.4)	11 (45.8)	81 (46.3)	0.96
No	81 (53.6)	13 (54.2)	94 (53.7)	
**Lung disease**, ***n*** **(%)**				
Yes	32 (21.2)	5 (20.8)	37 (21.1)	0.96
No	119 (78.8)	19 (79.2)	138 (78.9)	
**Renal failure**, ***n*** **(%)**				
Yes	48 (31.8)	5 (20.8)	53 (30.3)	0.28
No	103 (68.2)	19 (79.2)	122 (69.7)	
**Dyslipidemia**, ***n*** **(%)**				
Yes	72 (47.7)	12 (50)	84 (48)	0.83
No	79 (52.3)	12 (50)	91 (52)	
**Neurological illness**, ***n*** **(%)**				
Yes	32 (21.2)	5 (20.8)	37 (21.1)	0.96
No	119 (78.8)	19 (79.2)	138 (78.9)	
**Psychiatric illness**, ***n*** **(%)**				
Yes	10 (6.6)	1 (4.2)	11 (6.3)	0.64
No	141 (93.4)	23 (95.8)	164 (93.7)	

*PPMC, Padeh-Poriya Medical Center*.

* *Bold values indicate statistical significance*.

An analysis of the correlation between co-morbidities and mortality identified diabetes as a significant mortality risk factor, seen in 81 patients (46.2% of all patients) (*p* < 0.05), with no significant difference found between the carrier and clinical infection groups (data not shown). Other co-morbidities, such as ischemic heart disease, lung disease, showed no statistically significant correlation with mortality risk.

### Microbiological and Molecular Characteristics

Table 2 shows the different species of bacteria identified in carriers and clinically infected patients. In total, 189 isolates were cultured from 175 patients. *Klebsiella* spp. was the most prevalent bacteria in the carrier and the clinical infection groups (55.3 and 39.3%, respectively). *E. coli* was the second most prevalent bacteria in the carrier group (29.8%). *Enterobacter* spp. was more prevalent in the clinical infection group compared to the carrier group (35.7 vs. 11.2%, respectively). The least prevalent bacteria in both groups were *Citrobacter* spp. and *Providencia* spp. A significant association was noted between the duration of hospitalization and the type of CPE bacteria acquired, with a mean hospitalization duration of 14.9 days for *Klebsiella* spp., 19.2 days for *E. coli*, and 21.5 days for *Enterobacter* spp. (*p* < 0.01). In both groups, there was a predominance of bacteria carrying the KPC gene (Table 3). NDM was more prevalent in the clinical infection group (14.3 vs. 5%) while other resistance mechanisms were found exclusively in the carrier group (Table 3). Most (56.7%) KPC-positive bacteria were *K. pneumoniae*, while *Enterobacter* spp. constituted most of the (58.3%) NDM-positive bacteria (Table 4).

**Table 2:** Prevalence of different types of bacteria in CPE patient samples^*^.

**Bacteria**	**Bacteria from carriers** ** (*N* = 161)**	**Bacteria from clinical infection** ** (*N* = 28)**	**Total** ** (*N* = 189)**	***p*-value**
*E. coli*	48 (29.8%)	3 (10.7%)	51 (27%)	<0.001^**^
*Klebsiella* spp.	89 (55.3%)	11 (39.3%)	100 (52.9%)	
*Enterobacter* spp.	18 (11.2%)	10 (35.7%)	28 (14.8%)	
*Citrobacter* spp.	5 (3.1%)	3 (10.7%)	8 (4.2%)	
*Providencia* spp.	1 (0.62%)	1 (3.6%)	2 (1.06%)	

* *Data are presented as count and percent: n (%)*.

** *Bold values indicate statistical significance*.

**Table 3:** Distribution of CPE resistance mechanisms^*^.

**Resistance mechanism**	**Bacteria from carriers** ** (*N* = 161)**	**Bacteria from clinical infection** ** (*N* = 28)**	**Total** ** (*N* = 190)**	***p*-Value**
KPC	138 (85.71%)	24 (85.7%)	162 (85.7%)	0.12
NDM	8 (5.0%)	4 (14.3%)	12 (6.3%)	
OXA-48	13 (8.1%)	0	13 (6.9%)	
VIM	2 (1.2%)	0	2 (1.1%)	

* *Data are presented as count and percent: n (%)*.

**Table 4:** Distribution of CPE resistance mechanisms per species^*^.

**Resistance mechanism**	**KPC** ** (*N* = 162)**	**NDM** ** (*N* = 12)**	**OXA48** ** (*N* = 13)**	**VIM** ** (*N* = 2)**
*E. coli* (*N* = 51)	43 (26.5%)	2 (16.7%)	6 (46.15%)	0
*Klebsiella* spp. (*N* = 100)	92 (56.7%)	1 (8.3%)	6 (46.15%)	1 (50%)
*Enterobacter* spp. (*N* = 28)	19 (11.7%)	7 (58.3%)	1 (7.7%)	1 (50%)
*Citrobacter* spp. (*N* = 8)	8 (4.9%)	0	0	0
*Providencia* spp. (*N* = 2)	0	2 (16.7%)	0	0

* *Data are presented as count and percent: n (%)*.

The clinical isolates showed high aztreonam (82.14%), chloramphenicol (75%), and amikacin (64.3%) sensitivity rates (Table 5).

**Table 5:** Distribution of antibiotic susceptibility among clinical CPE isolates per carbapenemase type^*^.

**Antibiotic/Resistance mechanism**	**KPC** ** (*N* = 24)**	**NDM** ** (*N* = 4)**	**Total** ** isolates** ** (*N* = 28)**
Amikacin	16 (66.7)	2 (50)	18 (64.3)
Aztreonam	20 (83.3)	3 (75)	23 (82.14)
Ciprofloxacin	6 (25)	2 (50)	8 (28.6)
TMP-SMX	4 (16.6)	1 (25)	5 (17.8)
Fosfomycin	14 (58.3)	2 (50)	16 (57.1)
Gentamycin	5 (20.8)	0 (0)	5 (17.85)
Levofloxacin	12 (50)	1 (25)	13 (46.4)
Chloramphenicol	18 (75)	3 (75)	21 (75)

* *Data are presented as count and percent: n (%)*.

*TMP-SMX, trimethoprim/sulfamethoxazole*.

## Discussion

This study compared clinical and demographic characteristics of CPE carriers to those of patients with clinical CPE infections, in a primary care hospital in northern Israel. The study found a significantly higher percentage of community residents and nosocomial acquisitions and a higher mortality rate among patients with a new clinical infection with CPE as compared to CPE carriers with no clinical manifestations. To date, most studies on CPE have focused on clinically symptomatic patients and much less on asymptomatic carriers. Yet, the present study demonstrated that most subjects were CPE carriers without showing clinical signs of infection. These patients showed a higher rate of recent hospitalizations, and LTCF residency as compared to patients with an active infection.

While measures and guidelines differ between health care systems around the world (3, 10, 11), most reserve CPE screening to high-risk situations. Our medical center conducts much broader screening, as we described earlier. The presented results justify this strategy since 39.1% of the carriers acquired CPE during the current hospitalization and 56.3% of the carrier group had been hospitalized in the 3 months prior to the new CPE diagnosis. In addition, a significant portion of the patients (40.5%) were LTCF residents, which is consistent with reports singling LTCF out as a major risk factor for CPE acquisition (12, 13), but contradicts other works which classified CPE as almost exclusively healthcare facility-associated (10). The alarmingly large portion of CPE acquisitions occurring in the hospital, despite the strict isolation and disinfection protocols in place in the medical center, demand more vigilant adherence, both amongst medical staff and supporting staff, such as housekeeping and transportation.

The mortality rates in both the carrier and clinical infection groups were lower compared to the rates reported in previous studies, which were generally around 40% (14, 15). This discrepancy can be explained by the fact that most of the relevant literature focused on clinically symptomatic infections, while most of the subjects in the present study were asymptomatic carriers whose CPE diagnosis was irrelevant to their clinical presentation. The lower mortality rates in the clinical infection group as compared to the reported literature can be attributed to the large proportion of positive urinary cultures, whose clinical relevance is unknown; a 10% mortality rate was recorded during hospitalization or within 30 days of diagnosis in this subgroup of patients. In contrast, mortality during hospitalization among patients diagnosed via CPE bacteremia, stood at 50%, which is consistent with mortality rates reported in several studies (16, 17).

Examination of the distribution of bacterial species demonstrated that *Klebsiella* spp. was the most common bacteria in both carrier and clinical infection patients, followed by *E. coli* in the carrier group, and *Enterobacter* spp. in the clinical infection group. These findings align with reports from around the world showing an increased prevalence and associated mortality of *Enterobacter*-related sepsis, which was found in 50% of our bacteremia isolates, and was associated with 40–50% mortality rates (15, 18).

KPC proved to be the most prevalent resistance mechanism in the present sample set (85.1 and 85.7% in the carrier and clinical groups, respectively), which was consistent with other studies in Israel (15, 19), followed by NDM, OXA-48, and VIM. The emergence of NDM-positive isolates in carrier patients and more importantly, in the clinical infection isolates, raises concern since this resistance mechanism is not endemic in other countries in the region and, although it is spreading in Egypt and northern European countries, there have been no such reports in other countries in the Mediterranean basin and has only been reported sporadically in Israel (15). The isolation of VIM metallo-β-lactamase, which is endemic in Greece and is reportedly spreading in Italy and Spain, but rarely reported in Israel (15, 19), should serve as a warning sign, as this resistance mechanism (Ambler Class-B) (20) can spread to other hospitals in Israel and the region (15, 21).

Analysis of the antibiotic susceptibility profiles of the clinical isolates, identified sensitivity to aminoglycosides, a trend worth noting. Aminoglycosides are an important therapeutic option for resistant Gram-negative bacteria, most notably in the treatment of UTIs (22). The present work found that bacterial sensitivity to amikacin was substantially higher (64.28%) than to gentamicin (17.85%), possibly due the increasing use of gentamicin in the past decade as first-line empiric therapy. Indeed, most gentamicin-susceptible isolates were collected in the earlier portion of the study period, while amikacin-susceptible isolates remained relatively constant. These observations should prompt a discussion of the possible use of amikacin as empirical treatment for CPE patients, both in combination with other antibiotics for bacteremia and other severe infections, and as monotherapy for UTI (22).

In parallel, a high rate of chloramphenicol susceptibility (75% of clinical isolates) was recorded. The mostly bacteriostatic activity of chloramphenicol targets a wide spectrum of Gram-positive and Gram-negative bacteria. It is not in common use in the developed world, due to its side effects, including bone marrow suppression, aplastic anemia, gray-baby syndrome, and others, and is reserved for cases with limited alternatives, such as meningitis and brain abscesses, VRE bacteremia, and others. Nonetheless, it is still commonly used in the developing world (23). Clinical data and experience with chloramphenicol in the CPE settings is scarce, with only a few studies describing susceptibility, ranging between 7 and 35% (24, 25). The high rate of chloramphenicol susceptibility measured here should prompt design of studies testing its efficacy in treating CPE infections, either as monotherapy or as an adjunct agent alongside other first-line antibiotics [e.g., colistin; (26, 27)]. Other antibiotics assessed in the present study may be of clinical significance in the foreseeable future. Aztreonam, a monobactam to which 82.14% of isolates in our study were susceptible, is currently being tested in two different phase 3 trials in combination with avibactam, for treatment of various severe infections with limited therapeutic options (28). Conflicting reports on fosfomycin sensitivity exist, with some noting its potential in combination therapy or even as monotherapy, while others caution about high mortality rates when used intravenously (22, 28, 29). The fosfomycin sensitivity rate in the current study was 62.96%.

The current research had several limitations. It was a retrospective study in a single and small medical center in northern Israel that does not have post-transplantation units or a large surgical or trauma ICU unit. The study only targeted patients with a new diagnosis of CPE carriage or infection and excluded the majority of admitted patients with CPE whose carriage was already known from surveillance in LTCF and previous hospitalizations and who might have shifted the average demographic, clinical and/or microbiological profiles. These exclusion criteria also created a limited number of clinical isolates, in contrast to other works, which primarily focused on clinically symptomatic patients and not on asymptomatic carriers. The work also failed to differentiate between patients with urinary CPE-positive isolates but asymptomatic bacteriuria (most likely indicative of carriage than infection) and clinical UTI. The study also collected data on 30-day mortality, while 90-day mortality was not considered. Additionally, since antibiotic use is a main risk factor for CDI, we should have collected data regarding antibiotic exposure.

The findings in this study justify our prevention control strategy since 39.1% of the carriers acquired CPE during their hospitalization and 56.3% had been hospitalized in the 3 months prior to the new CPE diagnosis. Moreover, in light of the large proportion of CPE acquisitions occurring in the hospital, we should invest much effort in monitoring the implementation of our prevention strategy. The observations should be considered when redefining surveillance and treatment protocols for CPE infections.

## Data Availability Statement

The original contributions presented in the study are included in the article/supplementary material, further inquiries can be directed to the corresponding author/s.

## Ethics Statement

The studies involving human participants were reviewed and approved by this study received the approval of the Ethics Committee of the Poria Medical Center. In light of the fact that this is a study that includes data collection only the study received a sufficiently appropriate death exemption from the patients. Written informed consent from the participants' legal guardian/next of kin was not required to participate in this study in accordance with the national legislation and the institutional requirements.

## Author Contributions

AA, HZ, AP, and ON designed the study, analyzed, interpreted the data, and wrote the final manuscript. KL, IA, and MA were involved in development of the protocols. All authors read and approved the final manuscript.

## Conflict of Interest

The authors declare that the research was conducted in the absence of any commercial or financial relationships that could be construed as a potential conflict of interest.
